# Dissection of an old protein reveals a novel application: domain D of *Staphylococcus aureus *Protein A (sSpAD) as a secretion - tag

**DOI:** 10.1186/1475-2859-9-92

**Published:** 2010-11-23

**Authors:** Thomas Heel, Michael Paal, Rainer Schneider, Bernhard Auer

**Affiliations:** 1 Austrian Center of Industrial Biotechnology (ACIB), TU Graz, Petersgasse 14, A-8010 Graz, Austria; 2 Institute of Biochemistry, University of Innsbruck, Peter-Mayr-Strasse 1a, A-6020 Innsbruck, Austria; 3 Center for Molecular Biosciences (CMBI), University of Innsbruck, Peter-Mayr-Strasse 1a, A-6020 Innsbruck, Austria

## Abstract

**Background:**

*Escherichia coli *as a frequently utilized host organism for recombinant protein production offers different cellular locations with distinct qualities. The periplasmic space is often favored for the production of complex proteins due to enhanced disulfide bond formation, increased target product stability and simplified downstream processing. To direct proteins to the periplasmic space rather small proteinaceus tags that can be used for affinity purification would be advantageous.

**Results:**

We discovered that domain D of the *Staphylococcus aureus *protein A was sufficient for the secretion of various target proteins into the periplasmic space of *E. coli*. Our experiments indicated the Sec pathway as the mode of secretion, although N-terminal processing was not observed. Furthermore, the solubility of recombinant fusion proteins was improved for proteins prone to aggregation.

The tag allowed a straightforward affinity purification of recombinant fusion protein via an IgG column, which was exemplified for the target protein human superoxide dismutase 1 (SOD).

**Conclusions:**

In this work we present a new secretion tag that combines several advantages for the production of recombinant proteins in *E. coli*. Domain D of *S. aureus *protein A protects the protein of interest against N-terminal degradation, increases target protein solubility and enables a straight-forward purification of the recombinant protein using of IgG columns.

## Background

Due to the simple handling, inexpensive fast high-density cultivation and well-known genetics [1,2], *E. coli *remains an attractive host for the production of recombinant proteins, even though more complex proteins with posttranslational modifications, such as glycosylation patterns require alternative host systems [3,4]. Depending on the characteristics of the target protein, *E. coli *offers different compartments to meet the requirements for successful expression and purification. Cytoplasmic expression offers high yields of soluble product [5], but the purification from the cell lysate can be complex and costly. High level cytoplasmic overexpression may lead to the formation of inclusion bodies (IB). These protein aggregates simplify the purification but make *in vitro *refolding necessary [6,7].

In order to make purification easier, protect the target from degradation, (which is especially a problem with low molecular weight molecules [8]) or increase the chance of proper folding, the secretion of the target protein into the periplasm or the culture medium has proven to be a strong alternative [9,10]. Translocation of proteins across the inner membrane requires a signal peptide. However, the presence of a signal sequence alone does not ensure secretion into the periplasmic space [11,12]. Thus, a larger secretion moiety can be linked to the target gene. It has been shown that the *Staphylococcus aureus *Protein A (SpA) secretion signal combined with miscellaneous Protein A sub-domains directs heterologous proteins into the periplasm or even to the culture supernatant [13]. In addition to promoting protein translocation to the periplasm these domains have been shown to improve folding of the target protein and to protect against N-terminal degradation [14].

In the present work we show that domain D of SpA expressed from a synthetic codon optimized gene (sSpAD) is sufficient for the secretion of recombinant proteins. Furthermore we propose the Sec pathway mediating secretion and demonstrate the possibility of a straightforward one step expression and purification system.

## Results and discussion

**Figure 1:**
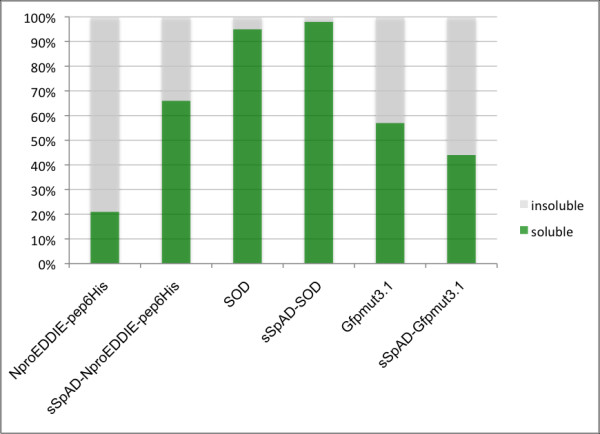
**Comparison of the protein solubility with and without sSpAD tag**. The solubility of N^pro^EDDIE-pep6His, SOD and GFPmut3.1 with and without sSpAD tag, expressed under the control of a LacUV5 promoter, was determined under defined cultivation conditions.

Preliminary experiments with the swine fever virus autoprotease N^pro^EDDIE [7] showed that protein solubility was drastically increased with a C-terminal sSpAD extension, whereas N^pro^EDDIE fusion proteins with other tags were deposited as insoluble aggregates within the cytoplasm [7,15]. Due to the characteristics of protein A as a surface protein it was assumed that an N-terminal sSpAD tag might facilitate periplasmic secretion. Therefore a construct with an sSpAD tag upstream of the autoprotease was cloned and this fusion protein was expressed under control of the weak lacUV5 promoter in *E. coli*. As shown in Figure 1 the tag enhanced the solubility of the aggregate forming protein N^pro^EDDIE-pep6His by a factor of three. The soluble fraction of human superoxide dismutase 1 (SOD), which is a highly soluble protein per se (95%), was enhanced only by three percent. However, solubility of GFPmut3.1 even decreased as a fusion with sSpAD. This can be explained by the observation that *E.coli *did not tolerate considerable amounts of GFPmut3.1 in the cytoplasm. We found the main portion of GFPmut3.1 either in the periplasm or in inclusion bodies (data not shown). In fusion with sSpAD the expression rate was increased even further. The additional amount of protein was mostly deposited in inclusion bodies and only a minor part exported to the periplasm (data not shown). This led to a slightly increased export but to an overall reduced solubility.

Subsequent isolation of the periplasmic fraction by osmotic shock treatment revealed the ability of the sSpAD moiety to mediate secretion. The different cell compartments were isolated according to the manual published by Paal et al. [16]. There are two main secretion pathways, known to mediate protein translocation across the inner membrane [17]. A number of specific inhibitors were used to distinguish between passive diffusion and active secretion by means of the Sec or Tat pathway.

### Identification of the secretion pathway

In order to identify the secretion pathway the fusion construct sSpAD-N^pro^EDDIE-pep6His was expressed under control of the strong T5 promoter. This promoter enabled the expression of detectable protein amounts in the presence of toxins, such as carbonyl cyanide m-chlorphenylhydrazone (CCCP) within short incubation times in different host strains.

**Figure 2:**
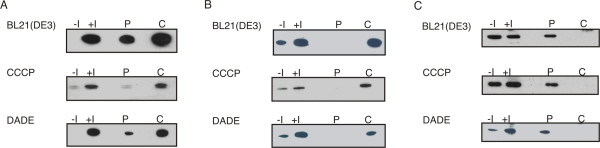
**Secretion competence of an sSpAD fusion protein**. A: The sSpAD tag reacts with most secondary antibodies. Therefore sSpAD fusion proteins were detected on the same membrane as GroEL immuno blots. For the sSpAD immuno blots a secondary antibody was used which reacts against sSpAD. B: Anti GroEL immuno blots of the isolated fractions were performed to confirm that the periplasmic fractions obtained via osmolysis, were not contaminated with cytoplasmic proteins. C: Anti MalE immuno blots were performed to confirm that the isolated fractions correspond with the periplasm as the Maltose Binding Protein E is a periplasmic protein. -I: whole cell sample without induction of recombinant protein expression, +I: whole cell samples with induction of recombinant protein expression, P periplasmic fraction, C: cytoplasmic fraction; CCCP: carbonyl cyanide m-chlorophenylhydrazone, BL21(DE3): host strain, DADE: ΔtatABCDΔtatE host strain derived from MC4100.

Several secretion pathways can be analyzed using diverse protonophores and knockout strains. CCCP has the ability to specifically inhibit all proton motive force driven pathways in *E. coli *[18,19]. To test if sSpAD secretion was driven by any of these pathways the recombinant fusion protein was expressed and cultivated in the presence of CCCP. As shown in Figure 2A, CCCP inhibited secretion into the periplasm. The slight signal in the periplasmic fraction could be considered as an artefact because minor expression of the recombinant protein occurs even before induction and addition of CCCP. Thus the signal in the periplasmic fraction was most likely due to secretion before inhibition of the given secretion pathway. Immuno blots against GroEL were performed to confirm the integrity of the spheroplasts. GroEL is a cytoplasmic chaperone, which was not found in the periplasm (Figure 2B). Anti Maltose binding protein E (MalE) immuno blots were performed to verify the isolated fractions as periplasm of *E. coli *cells (Figure 2C).

DADE is a MC4100 derived Tat knock-out strain lacking a functional Tat pathway[20]. This strain was used to show if sSpAD secretion was Tat dependent. Subcellular fractions showed that Tat knockout had no effect on the secretion capacity of the fusion protein shown in Figure 2A. Therefore, the involvement of the Tat pathway in the secretion process was excluded.

**Figure 3:**
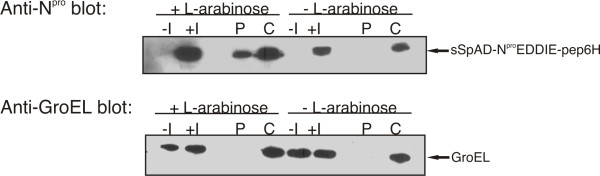
**Identification of the secretion pathway**. In the CM124 strain Sec mediated secretion is inducible via addition of L-arabinose. Incubation times were kept very short to ensure cell viability of the cell culture without functional Sec pathway. Furthermore, low expression levels of the recombinant protein prevent contamination of the subcellular compartments due to overexpression of the target protein. Therefore, the subcellular localization of N^pro^EDDIE was determined via immuno blot. GroEL immuno blot was performed to control osmolysis mediated cell lysis. -I: whole cell sample without induction of recombinant protein expression, +I: whole cell samples with induction of recombinant protein expression, P: periplasmic fraction, C: cytoplasmic fraction.

Sodium-azide is a strong inhibitor of the ATPase SecA and therefore has the ability to specifically inhibit the Sec translocation [21] in *E. coli*. Preliminary experiments with sodium-azide indicated an inhibition of translocation, although, recombinant expression artefacts were observed in the periplasmic fraction due to the osmotic shock procedure. Therefore, experiments were performed in parallel, using the target protein with and without the secretion tag to determine the background. The densitometric comparison of the amount of recombinant protein with and without sSpAD-tag in the periplasmic fraction confirmed that sodium-azide inhibited secretion. Continuative experiments were performed with the SecE knock-out strain CM124 [21]. As the Sec pathway is crucial for cell viability, SecE was complemented via expression from an L-arabinose induced plasmid. Over-night (including L-arabinose) culture was split, diluted and subsequently cultivated with and without L-arabinose. After dilution the culture without L-arabinose should have no functional Sec pathway. As shown in Figure 3 the knockout of the Sec pathway inhibited secretion of the target protein. These experiments led to the assumption that the Sec pathway seems to be involved in some way in the export of sSpAD fusion proteins to the periplasm; however the SignalP 3.0 prediction tool http://www.cbs.dtu.dk/services/SignalP did not reveal a canonical Sec secretion signal. The recent advances in genomics and proteomics revealed numerous extracellular proteins lacking defined secretion signals [22], often combining functions within the cytoplasm and in the extracellular environment [23]. Possibly, the elevated levels of expression of the sSpAD tagged proteins could lead to an emergency mechanism, which exports proteins through the Sec channel without using a signal sequence.

### Quantification of the secretion capacity

The fusions of sSpAD to the pestiviral autoprotease N^pro^EDDIE-pep6His, to green fluorescent protein GFPmut3.1 and to human superoxide dismutase 1 (SOD) produced by shaking flask cultivation were quantified by densitometry of SDS-PAGE as described in Methods. Since the fusion tag was not cleaved after secretion, it was not possible to distinguish easily between secreted and non-secreted proteins. Preliminary experiments showed that overexpressed proteins lacking export signals were detected in the periplasmic fraction. Therefore all recombinant proteins were expressed with and without sSpAD tag and the periplasmic fractions of all samples were isolated. Subsequently, the concentrations of the target proteins in the periplasm of all samples were measured and the amount found in the periplasm without sSpAD subtracted from the amount of proteins secreted with the sSpAD tag. The corrected secretion capacities are given in Table 1. The secretion yields for sSpAD-SOD (16.4 mg/L) and sSpAD-N^pro^EDDIE-pep6His (11.3 mg/L) were well above the reported yield for Sec mediated secretion [9]. However, in contrast to N^pro^EDDIE-pep6His (not detectable in the periplamic fraction) and SOD (approximately 30 percent in the periplamic fraction), a major part of GFPmut3.1 was found in the periplasm, although the lack of the cytoplasmic chaperone GroEL indicated intact spheroplasts. Due to expression of GFPmut3.1 from a codon-optimized gene the protein level of GFPmut3.1 was rather high and increased even with the sSpAD tag. Therefore it was assumed, that these high expression levels provoked a passive diffusion of GFPmut3.1 into the periplasm.

**Table 1:** Secretion capacity of the fusion proteins

Vectors	Fusion Protein	Secretion Capacity
pLacUV5^a^	sSpAD-N^pro^EDDIE-pep6His	11.3 mg/L

pLacUV5	sSpAD-Gfpmut3.1	1.3 mg/L

pLacUV5	sSpAD-SOD	16.4 mg/L

^a ^pET30a plasmids with the lacUV5 promoter are named pLacUV5

### One step secretion and purification

**Figure 4:**
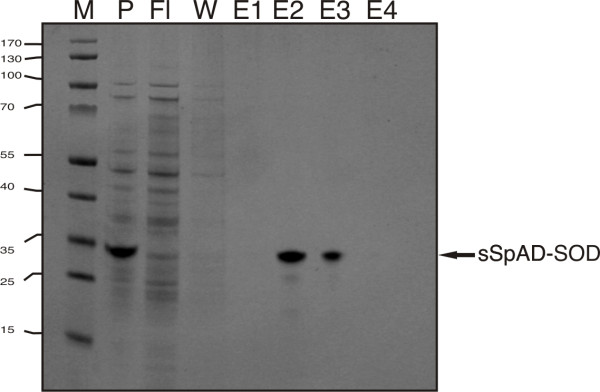
**IgG column purification of an sSpAD-fusion protein**. sSpAD-SOD was purified out of the periplasmic fraction of a shaking flask cultivation in BL21(DE3). P: periplasmic fraction, Fl: flow through, W: washing step, E1-E4: eluted fractions.

In order to exemplify the quick and easy purification of fusion proteins the periplasmic fraction of a 10 ml shaking flask cultivation of sSpAD-SOD was purified. The sample was applied on an IgG column as described in Methods. After a single purification step 16.9 mg/L of the purified fusion protein could be obtained (Figure 4). This was in good agreement to the amount found by determination of the secretion capacity (16,4 mg/L).

## Conclusions

The main advantage of this system is the applicability for a variety of different proteins and the improved yield of soluble product. Especially for aggregate forming proteins this tag provides an alternative to common solubility tags such as GST and MBP. In terms of secretion of heterologous targets, a typical Sec signal sequence is often not sufficient to promote the transport across the inner cell membrane. The sSpAD tag tends to enhance the solubility of aggregate forming fusion partners, which results in an improved secretion of the target protein. This was exemplified by the autoprotease N^pro^, a cystein rich protein, which does not secrete with a single Sec signal peptide (data not shown). Furthermore, the tag facilitates a straight-forward one step purification of the target protein, which was shown by the purification of sSpAD-SOD. Since sSpAD was not processed during Sec mediated secretion a proteolytic cleavage of the tag is necessary. Screening of sSpAD with the SignalP 3.0 prediction tool did not result in the detection of an intrinsic secretion signal. With an overall length of 7 kD the tag does not suit the classic Sec signal sequence. Therefore, it is proposed that sSpAD is not cleaved by the signal peptidase and possibly activates the SecA translocation by its conformation. Further dissection of sSpAD may identify an intrinsic secretion signal, which still facilitates an affinity-mediated purification.

## Methods

All experiments were performed with Milli-Q ultrapure water (Millipore purification system). *E. coli *cells were cultivated in TY growth medium supplemented with 50 μg/ml kanamycin and/or 100 μg/ml ampicillin depending on the used plasmid. For recombinant plasmid isolation *E. coli *K12 DH5α strain was used, whereas protein expression was performed in BL21(DE3), CM124 (ΔSecE with SecE under control of the araBAD promoter placed on a plasmid) and MC4100-DADE (ΔtatABCDE).

Restriction enzymes, GoTaq^® ^DNA polymerase, including the PCR buffer, were obtained from Promega. Molecular mass standard used for SDS-PAGE, rapid DNA ligation kit, Pfu DNA polymerase and 10x MgSO_4_-PCR buffer were obtained from Fermentas. Tris-Glycine gels were purchased from Invitrogen. Protran BA 83 nitrocellulose membrane was obtained from Whatman. Mouse anti-GroEL monoclonal antibody was purchased from Stressgen Bioreagents, Goat anti-Mouse IgG (HRP conjugated) from Invitrogen, anti-Maltose Binding Protein (MBP) monoclonal antibody (HRP conjugated) and anti-Maltose Binding Protein (MBP) polyclonal antibody from New England Biolabs. Anti-N^pro ^antibody was generated within the Austrian Center of Biopharmaceutical Technology at the BOKU Vienna. Syringe filters (pore size 0.45 μm) were from Sartorius, 10 kDa molecular weight cut-off ultrafiltration devices (Centriprep Ultracel YM-10 tubes, series 8000 stirred cell including Ultracel YM-10 membranes) from Millipore. The BCA™ protein assay kit was obtained from Pierce.

### Construction of expression plasmids

The pET30a T7 promoter (T7p) was replaced by three alternative promoters e.g. T5, the artificial Tac promoter and the lacUV5 promoter resulting in the three vectors pT5, pTac and pLacUV5. Two oligonucleotides corresponding to the given promoter sequence with complementary bases, prom lacUV5 SphI F and prom lacUV5 XbaI R, were directly ligated into an SphI and XbaI digested pET30a plasmids. The Tac promoter, consisting of two oligonucleotides with 65 complementary bases, prom Tac SphI F and prom Tac XbaI R, was directly ligated into SphI and XbaI digested pET30a plasmids. The T5 promoter was generated by PCR with the given primers in Table 2 and subsequently cloned into SphI and XbaI digested pET30a plasmids.

**Table 2:** Oligonucleotides used in this study

Primers	Sequences (5'- 3')
pET 30 sSpAD NdeI F	GCACGACATATGGCAGACGCACAACAGAATAAG

pET 30 sSpAD NdeI R	TAGCAGCATATGTTTTGGTGCCTGGAGTTC

pLacUV5 sSpAD NheI R	GCAAGCTAGCTTTTGGTGCCTGAGATTCGTTC

SOD NheI F	TAAAGCTAGCGCGGCAACAAAGGCCGTGTG

SOD SalI R	AGTTGTCGAC TTGGGCGATCCCAATTACACC

sGFP F NdeI	GGATCCACTCATATGAGCAAAGGCGAAG

sGFP R	CGAGGTCGACTTATTATTTATACAGTTCATC

prom Tac SphI F	**5'P**-CGAGCTGTTGACAATTAATCATCGGCTCGTATAATGTGTGGAATTGTGAGCGGATAACAATTT

prom Tac XbaI R	**5'P-**CTAGAAATTGTTATCCGCTCACAATTCCACACATTATACGAGCCGATGATTAATTGTCAACAGCTCGCATG

prom LacUV5 SphI F	**5'P**-CCCAGGCTTTACACTTTATGCTTCCGGCTCGTATAATGTGTGGAATTGTGAGCGGATAACAATTT

prom LacUV5 XbaI R	**5'P**-CTAGAAATTGTTATCCGCTCACAATTCCACACATTATACGAGCCGGAAGCATAAAGTGTAAAGCCTGGGCATG

T5 Prom SphI F	GGCGGCATGCGAAATCATAAAAAATTTAT

T5 Prom XbaI R	TTTCTAGATGTGTGAAATTGTTATCCGCT

^a ^The oligonucleotides contain the letters: F - forward or R - reverse

**5'P **represents a 5'Phosphate group modification

Codon optimized GFPmut3.1 gene was amplified using the primers sGFP F NdeI start and sGFP R (Table 2). The amplified fragment was ligated into NdeI and SalI digested pLacUV5 vector, resulting in pLacUV5-GFPmut3.1 plasmid.

The sSpAD signal sequence was codon optimized (sequence given in the appendix 1) and amplified using the primers pET30 sSpAD NdeI F and pET30 sSpAD NdeI R. Subsequently sSpAD was subcloned into the pLacUV5 GFPmut3.1 vector resulting in the pLacUV5-sSpAD-GFPmut3.1.

The sSpAD-N^pro^-EDDIE-pep6His construct was generated by digestion with NdeI of the pET30 N^pro^-EDDIE-pep6His vector and subsequent ligation with the same insert generated for the pLacUV5-sSpAD-GFPmut3.1 construct. Subsequently the promoter of the pET30-sSpAD-N^pro^-EDDIE-pep6His construct was replaced with the promoter LacUV5.

The pLacUV5-sSpAD-SOD plasmid was generated in two steps. First sSpAD was amplified using the primers pET 30 sSpAD NdeI F and pLacUV5 sSpAD NheI R and cloned into the pLacUV5 vector, which resulted in the pLacUV5 vector with an additional NheI restriction site. Subsequently the codon optimized SOD gene was amplified using the primers SOD NheI F and SOD SalI R (Table 2). The amplified fragment was ligated into the pLacUV5-sSpAD-Nhe vector, which was digested with NheI and SalI, resulting in pLacUV5 sSpAD-SOD plasmid. A list of the constructs is given in Table 3.

**Table 3:** Plasmids and corresponding expression products

**Vectors**^**a,b,c**^	Gene cloned	Resulting vectors	Expression product
pT5 ^b^	sSpAD-N^pro^EDDIE-pep6His	pT5 sSpAD-N^pro^EDDIE-pep6His	sSpAD-N^pro^EDDIE-pep6His

pTac ^c^	sSpAD-N^pro^EDDIE-pep6His	pTac sSpAD-N^pro^EDDIE-pep6His	sSpAD-N^pro^EDDIE-pep6His

pLacUV5 ^a^	sSpAD-Gfpmut3.1	placUV5 sSpAD-Gfpmut3.1	sSpAD-Gfpmut3.1

pLacUV5	sSpAD-SOD	placUV5 sSpAD-SOD	sSpAD-SOD

^a ^pET30a plasmids with the lacUV5 promoter are named pLacUV5

^b ^pET30a plasmids with the T5 promoter are named pT5

^c ^pET30a plasmids with the Tac promoter are named pTac

### Shake flask cultivation

Expression plasmids were transformed into *E. coli *BL21(DE3), subsequently a single colony was picked to inoculate overnight cultures. These cultures were diluted 1:20 with fresh TY-medium, supplemented with 0.5% glucose and grown to a density of OD_600_: 1.0 at 37°C/225 rpm. Recombinant protein synthesis was induced adding 1 mM IPTG. Expression with T5 promoter was carried out for 1 h at 37°C/225rpm in BL21(DE3), BL21(DE3) with 2 mM NaN_3_, BL21(DE3) 50 μM CCCP. For the shake flask cultivation with *E. coli *DADE experimental procedure was similar to *E. coli *BL21(DE3) but without addition of glucose during cultivation.

In contrast overnight cultures of CM124 cells carrying pET30-Tac-promoter-plasmids were grown in the presence of 0.2% L-arabinose. These overnight cultures were diluted 1:20 and split. The divided cultures were grown with and without 0.2% L-arabinose in parallel. Expression was induced at OD_600_: 0.5 with 1 mM IPTG and the cultures were incubated for 1 h at 37°C/225 rpm.

For the determination of the solubility and the secretion capacity overnight cultures of the host strain BL21(DE3) carrying the pET30-LacUV5-promoter plasmids were diluted 1:20. Expression was induced at OD_600_: 0.5 with 1 mM IPTG and the cultures were incubated for 2 h at 37°C/225 rpm.

### Cell fractionation

Isolation of the periplasm was performed at 24°C with a gentle osmotic shock procedure to minimize *E. coli *cell disruption during preparation (modified from [15]). After expression, a culture volume corresponding to 10 ml of OD_600 _2.0 was centrifuged at 3000 g for 10 min. The pellet was completely suspended in one culture volume of osmolysis buffer (100 mM TRIS-HCl pH 7.8, 15.4% sucrose, 3 mM EDTA) and incubated at 50 rpm for 10 min, followed by centrifugation. The supernatant was discarded and the pellet was resuspended in one culture volume water and incubated for 10 min at 50 rpm. Afterwards the suspension was centrifuged for 10 min at 3000 g. The supernatant, containing periplasmic proteins, was decanted and filtrated (membrane, 0.45 μm pore size). The spheroplasts were kept as a cellular fraction. For SDS-PAGE analysis periplasmic and cytoplasmic fraction samples were precipitated with TCA and all pellets solubilised in loading buffer (62.5 mM Tris-HCl pH 6.8, 10% glycerol, 2% SDS, 0.0025% bromophenol blue, 50 mM DTT).

For the determination of the solubility the cells were taken up in a culture volume lysis buffer (20 mM Na_2_HPO_4 _pH 8.0, 75 mM NaCl, 5 mM EDTA) and disrupted with a French press (American Instruments Co., Inc). Aliquots of the lysate were collected and centrifuged at 14000 rpm for 15 min. The supernatant contained soluble cytoplasmic protein, whereas the pellet represented the insoluble protein fraction. For SDS-PAGE analysis the samples were precipitated with TCA and all pellets solubilized in loading buffer (62.5 mM Tris-HCl pH 6.8, 10% glycerol, 2% SDS, 0.0025% bromophenol blue, 50 mM DTT).

### Concentration and purification of recombinant protein

To the filtrated periplasm fraction Na_2_HPO_4_/NaH_2_PO_4 _and NaCl were added to a final concentration of 20 mM Na_2_HPO_4_/NaH_2_PO_4, _500 mM NaCl, pH 8. 10 ml of this solution were concentrated to 2 ml with ultrafiltration devices. After removal of precipitated proteins by centrifugation, the supernatant was applied to a pre-equilibrated 500 μl gravity flow IgG-NHS-Sepharose column and purified. The column was washed with 2.5 ml 20 mM Na_2_HPO_4_/NaH_2_PO_4 _500 mM NaCl pH 8 buffer. The recombinant protein was eluted with 5 column volumes of 0.2 glycine buffer pH 3. The eluted fractions were pooled and the total concentration of the purified protein was quantified.

### Immuno blots

Cellular integrity after expression of the recombinant proteins and subsequent osmotic shock treatment was surveyed by Immuno blot analysis with antibodies against the periplasmic Maltose binding protein MalE, and the cytoplasmic chaperone GroEL. Cell fraction samples were separated on 4-20% Tris-Glycine gels and the proteins electrophoretically transferred onto nitrocellulose membranes. Incubation times of the antibodies were carried out according the instruction manuals.

### Protein quantification

Quantification of the secretion capacity was carried out by densitometric analysis of target proteins in comparison to BSA standards on Coomassie stained gels. Gels were photographed and analyzed with AlphaEaseFC software (Alpha Innotech Corporation).

## Abbreviations used

sSpAD: *Staphylococcus aureus *Protein A domain D expressed from a synthetic (codon optimized) gene; BL21(DE3): host strain; CCCP: carbonyl cyanide m-chlorophenylhydrazone; DADE: host strain derived from MC4100 ΔtatABCD, ΔtatE; DTT: dithiothreitol; EDTA: ethylene-diamine-tetra-acetic acid; GroEL: chaperone heat shock protein 60; GFP: Green fluorescent protein; IPTG: isopropyl-β-D-thiogalactopyranoside; MalE: maltose binding protein E; NaN_3: _sodium-azide; PCR: polymerase chain reaction; SDS: sodium dodecyl sulphate; SOD: Human Superoxide Dismutase 1; TCA: trichloro acetic acid;

## Competing interests

The authors declare that they have no competing interests.

## Authors' contributions

TH performed the experiments and wrote the manuscript. MP was involved in cloning the constructs and writing the manuscript. RS and BA were involved in the design of the experiments. All authors participated in editing the manuscript and all have read and approved the final version.

## Appendix 1

### Accession numbers

GFPmut3.1 [P42212]; SOD [BT008028.1]; Staphylococcal Protein A[P38507]:Domain D:

ADAQQNKFNKDQQSAFYEILNMPNLNEEQRNGFIQSLKDDPSQSTNVLGEAKKLNESQAPK N^pro^EDDIE [7]

DNA sequence sSpAD (codon optimized):

GCAGACGCACAACAGAATAAGTTTAACAAAGACCAGCAGAGCGCATTCTACGAAATTCTGAACAT

GCCGAATCTGAATGAGGAACAACGTAATGGCTTTATTCAGTCTTTAAAAGACGACCCATCTCAGA

GCACCAACGTTCTGGGCGAAGCAAAGAAACTGAACGAATCTCAGGCACCAAAA
